# Non-coding stem-bulge RNAs are required for cell proliferation and embryonic development in *C. elegans*

**DOI:** 10.1242/jcs.166744

**Published:** 2015-06-01

**Authors:** Madzia P. Kowalski, Howard A. Baylis, Torsten Krude

**Affiliations:** Department of Zoology, University of Cambridge, Downing Street, Cambridge, CB2 3EJ, UK

**Keywords:** Non-coding RNA, Cell proliferation, DNA replication

## Abstract

Stem bulge RNAs (sbRNAs) are a family of small non-coding stem-loop RNAs present in *Caenorhabditis* *elegans* and other nematodes, the function of which is unknown. Here, we report the first functional characterisation of nematode sbRNAs. We demonstrate that sbRNAs from a range of nematode species are able to reconstitute the initiation of chromosomal DNA replication in the presence of replication proteins *in vitro*, and that conserved nucleotide sequence motifs are essential for this function. By functionally inactivating sbRNAs with antisense morpholino oligonucleotides, we show that sbRNAs are required for S phase progression, early embryonic development and the viability of *C. elegans in vivo*. Thus, we demonstrate a new and essential role for sbRNAs during the early development of *C. elegans*. sbRNAs show limited nucleotide sequence similarity to vertebrate Y RNAs, which are also essential for the initiation of DNA replication. Our results therefore establish that the essential function of small non-coding stem-loop RNAs during DNA replication extends beyond vertebrates.

## INTRODUCTION

Small non-coding RNAs, which are less than 200 nucleotides in length, are involved in a multitude of cellular processes. These non-coding RNAs include small nucleolar (sno)RNAs that modify pre-rRNA, small nuclear (sn)RNAs that are involved in pre-mRNA splicing, micro (mi)RNAs that regulate mRNA translation and stability, and PIWI-interacting (pi)RNAs that mediate epigenetic and post-transcriptional gene silencing (reviewed by Morris and Mattick, 2014). More recently, other non-coding RNAs have emerged, and these have important roles in genome stability. Several small non-coding RNAs have been implicated in the control of DNA replication (Christov et al., 2006; Christov et al., 2008; Collart et al., 2011; Gardiner et al., 2009; Krude et al., 2009) and the DNA damage response in vertebrates (Chowdhury et al., 2013; Sharma and Misteli, 2013).

Stem-bulge RNAs (sbRNAs) are a recently identified family of non-coding RNAs found in nematode worms (Aftab et al., 2008; Boria et al., 2010; Deng et al., 2006). The genome of *Caenorhabditis elegans* encodes at least 19 different sbRNAs, each with an individual putative RNA polymerase III promoter (Boria et al., 2010). Although there have been expression level studies (Aftab et al., 2008; Boria et al., 2010; Deng et al., 2006), no function has been described for nematode sbRNAs to date.

Based on conserved nucleotide sequence elements and structural motifs, it has been suggested that sbRNAs might be homologues of vertebrate Y RNAs (Boria et al., 2010). Both sbRNAs and Y RNAs share an overall stem-loop structure containing a bulged double-stranded stem and an internal single-stranded loop of varying length and nucleotide sequence. In both species, the stem is divided into an upper section containing a highly conserved A/GUG-CAC/U motif and a lower section containing a single-stranded bulged cytosine. The 5′ terminus is base-paired to the 3′ end, which extends into a short a poly(U) tail (Boria et al., 2010).

Several independent biological functions have been described for Y RNAs (reviewed by Hall et al., 2013). Y RNAs can associate, through their lower stem and tail domains, with Ro60 (also known as TROVE2) and La (SSB) proteins to form Ro ribonculeoprotein complexes (Ro RNPs) (Hendrick et al., 1981; Lerner et al., 1981). Ro RNPs are involved in RNA quality control, RNA stability and cellular response to stress in several species (reviewed by Chen and Wolin, 2004; Hall et al., 2013; Wolin and Cedervall, 2002).

Y RNAs are also essential for the initiation of chromosomal DNA replication in vertebrates (Christov et al., 2006; Christov et al., 2008; Collart et al., 2011; Gardiner et al., 2009; Krude et al., 2009; Langley et al., 2010). Y RNAs were biochemically purified from human cell extracts in a functional screen for components that are essential in order to reconstitute chromosomal DNA replication in a cell-free system (Christov et al., 2006). In this system, chromosomal DNA replication initiates in nuclei isolated from late G1 phase human cells, when they are incubated in a cytosolic cell extract from proliferating cells *in vitro* (Krude, 2000). Specific depletion of Y RNAs from the proliferating cell extract inhibits the initiation step of DNA replication (Christov et al., 2006; Gardiner et al., 2009; Krude et al., 2009). The initiation function of the depleted extract is restored by the addition of any human or vertebrate Y RNA synthesised *in vitro*, showing that vertebrate Y RNAs are functionally redundant with each other in this system. This redundancy is due to the presence of an essential and sufficient domain in the upper stem of all vertebrate Y RNAs that includes a conserved GUG-CAC nucleotide sequence motif (Gardiner et al., 2009). Mutations of this sequence abolish the ability of the Y RNA to support DNA replication initiation and lead to structural disruption of this domain (Gardiner et al., 2009; Wang et al., 2014).

Vertebrate Y RNAs are also essential for DNA replication *in vivo*. RNA interference (RNAi) against Y RNAs in proliferating cultured vertebrate cells inhibits DNA replication and cell proliferation (Christov et al., 2006; Christov et al., 2008; Collart et al., 2011; Gardiner et al., 2009). Furthermore, a direct functional depletion of Y RNAs using antisense morpholino oligonucleotides (MOs) in embryos of the amphibian *Xenopus laevis* or the zebrafish *Danio rerio* leads to a dominant-negative inhibition of DNA replication, arrested development and early embryonic death just after the mid-blastula transition (Collart et al., 2011).

Although the role of Y RNAs in vertebrates is becoming clearer, their characteristics and roles in non-vertebrates are less clear. A non-vertebrate Y RNA (CeY) has been described in *C*. *elegans* and been shown to form Ro RNPs by binding to the nematode Ro protein homologue, ROP-1 (Labbé et al., 2000; Labbé et al., 1999; Van Horn et al., 1995). CeY RNA is not essential, as worms with the CeY gene deleted are viable (Boria et al., 2010). CeY RNA does not have sequence similarity to vertebrate Y RNAs in the upper stem domain and is unable to substitute for vertebrate Y RNAs in DNA replication assays *in vitro* (Boria et al., 2010; Gardiner et al., 2009), indicating that it does not fulfil the role of Y RNAs in DNA replication. Thus, it was hypothesised that this role could be fulfilled by the Y-RNA-related family of sbRNAs (Boria et al., 2010).

In this study, we report the first functional characterisation of nematode sbRNAs. We find that sbRNAs from *C. elegans* and a range of other nematode species are able to reconstitute chromosomal DNA replication *in vitro.* This activity is dependent on key structural RNA domains that are conserved between sbRNAs and vertebrate Y RNAs. We show, by functionally inactivating sbRNAs with antisense MOs, that sbRNAs are essential for viability, early embryonic development and normal S phase progression of *C. elegans in vivo*. We thus demonstrate a new and essential role for sbRNAs during the early development of *C. elegans*.

## RESULTS

### Conservation of nucleotide sequence and secondary structure predictions of *C. elegans* sbRNAs

**Fig. 1.**
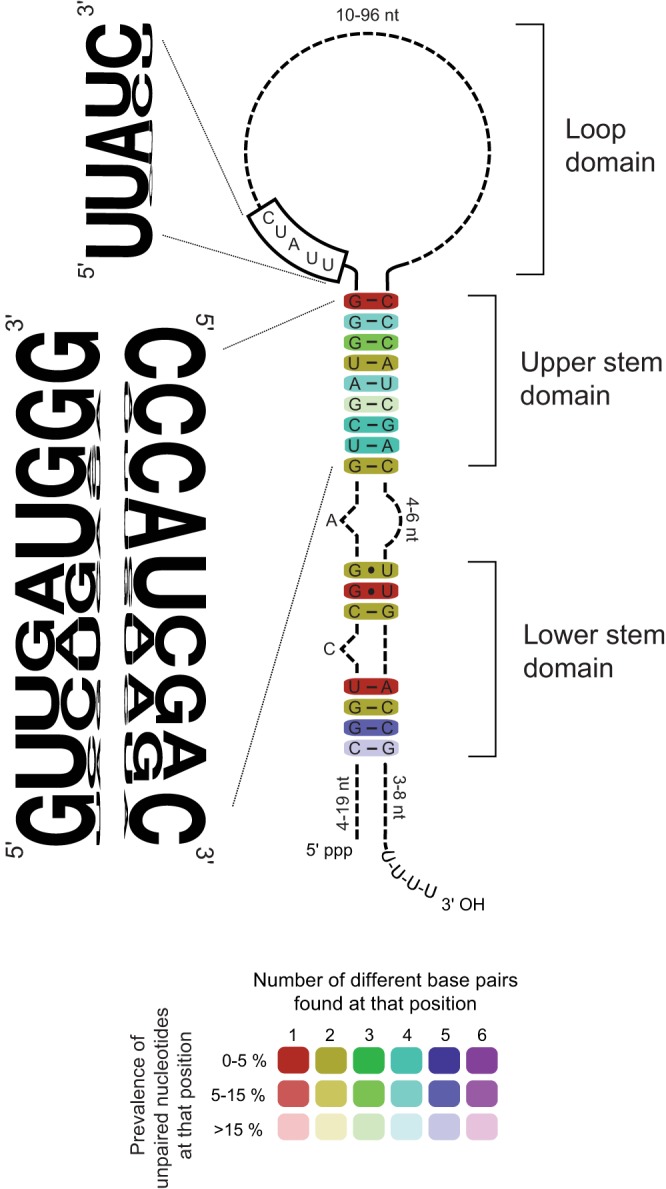
**Consensus secondary structure and nucleotide sequence of sbRNAs in *C. elegans.*** The consensus sbRNA was derived from manual sequence alignment of the 19 sbRNAs and is based on secondary structure predictions by MFold and LocARNA. Conserved structural domains are labelled. For the double-stranded domains, the most frequent base pair found at that position is displayed. The extent of nucleotide sequence conservation and structural conservation is indicated for each base pair by colour coding; the colour illustrates the extent of sequence conservation for each base pair by indicating the number of different base pairs found at that position in the predicted structure. The colour saturation indicates the structural conservation of each base pair, decreasing in saturation as the number of unpaired nucleotides found at that position increases. Frequencies of nucleotides in the evolutionary conserved domains of the double-stranded upper stem and in the single-stranded loop motif are illustrated by WebLogos.

To investigate the functionality of sbRNAs, we focused on the 19 family members in the model organism *C. elegans*, including the derived CeY RNA. By manually aligning all 19 sbRNA sequences, we derived a *C. elegans* consensus sbRNA in terms of nucleotide sequence and predicted secondary structure (Fig. 1).

The lower double-stranded stem domain of *C. elegans* sbRNAs, which is interrupted by a single bulged cytosine, is the most highly conserved domain, with five out of seven base pairs being either absolutely conserved, or allowing for two alternate base pairs (Fig. 1). The distinct upper stem domain consists of nine base pairs, with near-perfectly conserved GC clamps at either end, suggesting that the ability of this region to form a stable double-stranded stem is important. The nucleotide sequence in-between the GC clamps is also highly conserved, in particular a central UG-CA tetra-nucleotide motif. Notably, these features are also conserved in the upper stem domain of vertebrate Y RNAs (Wang et al., 2014), but not in the previously reported CeY RNA (Boria et al., 2010). Finally, the central loop domain remained unaligned owing to high sequence variation; however, it contained a highly conserved sequence motif (UUAUC) at its 5′ end (Fig. 1). Taken together, these three structural domains of the *C. elegans* consensus sbRNA are consistent with the overall consensus derived from all nematode sbRNAs and are highly similar to the corresponding domains in vertebrate Y RNAs (Boria et al., 2010; Wang et al., 2014).

### sbRNAs support the initiation of chromosomal DNA replication in a cell-free system

Vertebrate Y RNAs are essential for the initiation of chromosomal DNA replication (Christov et al., 2006; Collart et al., 2011; Gardiner et al., 2009; Krude et al., 2009; Langley et al., 2010). Given the sequence and structure homology between sbRNAs and vertebrate Y RNAs (Boria et al., 2010), we tested whether sbRNAs are also functional homologues of vertebrate Y RNAs.

**Fig. 2.**
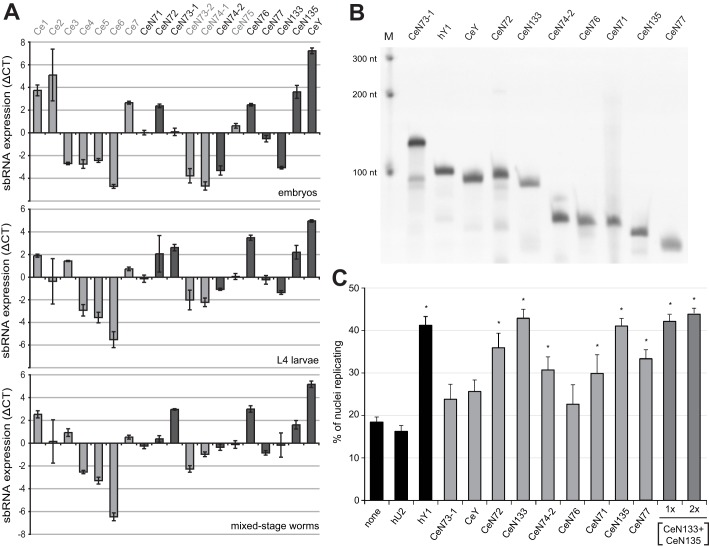
**sbRNAs support the initiation of DNA replication *in vitro*.** (A) Relative sbRNA expression levels. Total RNA was isolated from *C. elegans* embryos, synchronised L4 larvae and mixed-stage worms, and the relative sbRNA expression levels were determined by quantitative RT-PCR (top, middle and bottom panels, respectively). Binary log values of the mean±s.e.m. from three biological replicates (*n*=3) are shown as differences in threshold cycles (ΔCT) between the individual sbRNA and the overall mean of all sbRNAs (ΔCT=CT_sbRNA_–CT_mean_) after normalisation to a genomic DNA control. Dark grey highlighting indicates those sbRNAs that were selected for further analysis as detailed below. (B) Synthesis of sbRNAs. A selection of sbRNAs were synthesised by *in vitro* transcription, purified and visualised using denaturing gel electrophoresis and staining with SYBR Gold. Human Y1 (hY1) RNA was used as a reference. Markers (M) are RNA oligonucleotides of the indicated lengths. (C) A subset of sbRNAs can substitute for Y RNAs in a cell-free DNA replication initiation system. Template nuclei from late G1 phase cells were incubated with protein fractions QA and ArFT and the indicated RNAs. Human U2 snRNA (hU2) and hY1 RNA served as negative and positive controls, respectively. A standard overall concentration of 170 nM of each RNA per reaction were used, 2x indicates twice as much. Proportions of replicating nuclei were determined by immunofluorescence microscopy. Mean±s.e.m. values are shown for 3–25 experiments (*n*=3–25). *P<0.05 when compared to background level with no RNA added, as determined by Student's *t*-tests.

We first determined the relative expression levels of *C. elegans* sbRNAs during development by quantitative reverse-transcription PCR (qRT-PCR) (Fig. 2A). We determined the relative expression levels for 18 *C. elegans* sbRNAs and CeY RNA in embryos, L4 larvae and in a mixed-stage worm population. The overall pattern of relative sbRNA expression showed only small changes during development. CeY, CeN76, CeN135 and Ce1 RNAs were expressed at the highest levels, whereas Ce6, Ce5 and CeN73-2 RNAs had the lowest expression levels (Fig. 2A). The relative proportion of Ce2 among the sbRNAs decreased during development, whereas those of Ce3, CeN73-1, CeN74-1, CeN74-2 and CeN133 increased.

We then selected nine sbRNAs for further functional testing (those highlighted in dark grey in Fig. 2A). We synthesised and purified the sbRNAs *in vitro* (Fig. 2B) and tested whether they could initiate chromosomal DNA replication in an established cell-free system (Fig. 2C). In this system, late G1-phase template nuclei from human cells initiate chromosomal DNA replication upon incubation in a cytosolic extract from proliferating human cells, which contains DNA replication factors and endogenous Y RNAs (Christov et al., 2006; Krude, 2000). In order to test for the function of exogenous RNAs in this system, the endogenous Y RNAs are removed from the cell extract by biochemical fractionation (Christov et al., 2006). This yields two protein fractions, termed QA and ArFT, containing all essential soluble DNA replication proteins, but lacking the endogenous Y RNAs. Exogenous RNAs are then added to the two protein fractions and the proportions of replicating nuclei are scored by confocal immunofluorescence microscopy (Christov et al., 2006; Gardiner et al., 2009). When incubated in fractions QA and ArFT alone, 18% of template nuclei replicated (Fig. 2C). These nuclei represent contaminating S phase nuclei amongst the G1 phase template nuclei (Christov et al., 2006; Krude, 2000). Addition of human Y1 RNA as a positive control increased the proportion to ∼40%. Upon addition of purified *C. elegans* sbRNAs, six out of nine sbRNAs significantly increased the proportion of nuclei replicating over and above the background level of the negative control U2 snRNA (Student's *t*-test, *P*<0.05, Fig. 2C). A combination of CeN133 and CeN135 sbRNA together was as effective as the same amount of either sbRNA alone, and doubling the amount did not increase the proportion of nuclei replicating further (Fig. 2C). In contrast, CeY RNA did not significantly increase the proportion of nuclei replicating (Student's *t*-test, *P*>0.05), as previously reported (Gardiner et al., 2009). We conclude that some, but not all, sbRNAs are able to support chromosomal DNA replication *in vitro* and act redundantly with each other.

Next, we examined whether this functional conservation in DNA replication *in vitro* extends to other nematode sbRNAs. Using the same approach, we investigated sbRNAs from four other nematode species (Boria et al., 2010): *Haemonchus contortus*, *Pristionchus pacificus*, *Meloidogyne hapla* and *Meloidogyne incognita* (supplementary material Fig. S1A). We selected representative sbRNAs from these species based on their secondary structure, the presence of recognisable promoters (Boria et al., 2010) and the presence of structural motifs in the loop and upper stem domains within the same species (supplementary material Fig. S1B). All four sbRNAs increased the proportion of replicating nuclei to between 30–40% (supplementary material Fig. S1C), significantly above the background level (Student's *t-*test, *P*<0.01). Thus, sbRNAs from species across the phylum Nematoda can substitute for human Y RNAs in the initiation of chromosomal DNA replication *in vitro*.

### Functional activity of sbRNAs is dependent on conserved RNA domains

**Fig. 3.**
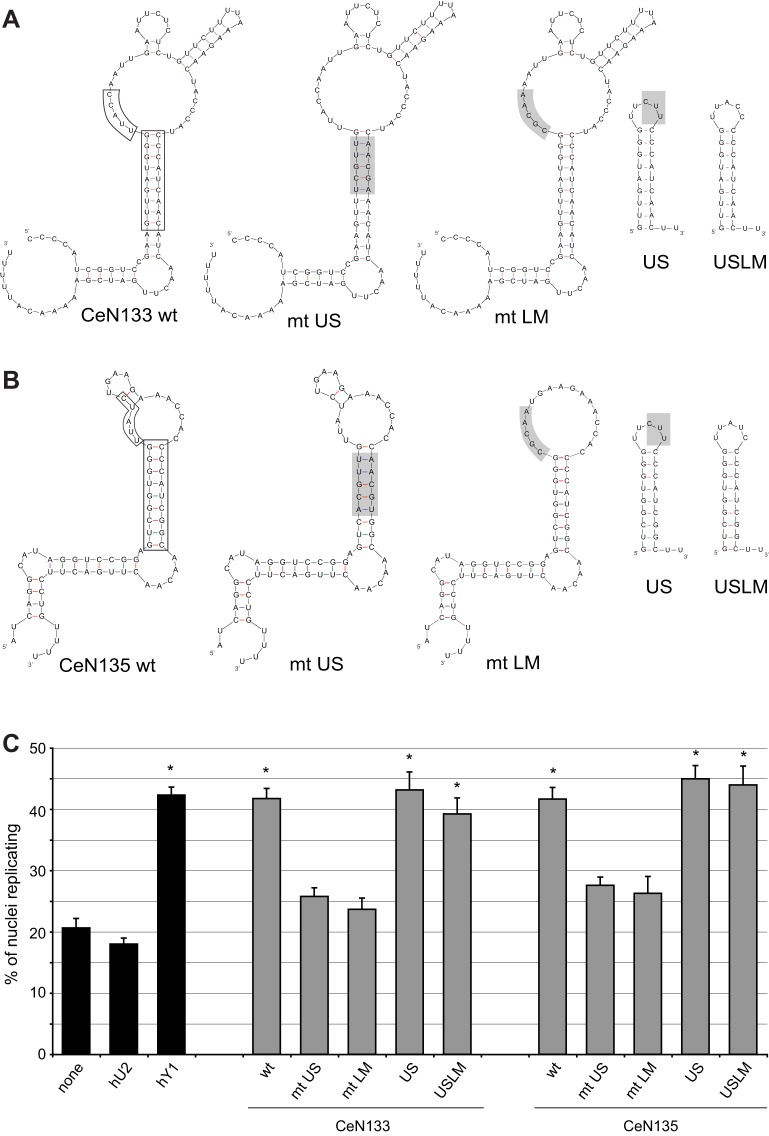
**Mutating conserved domains of sbRNAs compromises DNA replication *in vitro.*** Nucleotide sequences and predicted secondary structures of wild-type (wt) and mutant (mt) sbRNAs. (A) CeN133 sbRNAs. (B) CeN135 sbRNAs. Conserved upper stem and loop domains are boxed on the wt RNA. Mutant sbRNAs were synthesised with substitutions in either the upper stem (mt US) or loop motif (mt LM). Short stem loop RNAs comprising the upper stem linked either with an internal penta-pyrimidine loop (US) or the conserved 5′ loop motif of the corresponding wt sbRNA (USLM) were also tested. The mutated motifs are shaded. (C) The indicated wild-type and mutant sbRNAs were tested for their ability to initiate DNA replication as detailed for Fig. 2C (mean±s.e.m.; *n*=3–8). *P<0.05 when compared to background level with no RNA added, as determined by Student's *t*-tests.

Next, we determined which structural domains are important for sbRNA function *in vitro*. We have shown previously that mutations in conserved sequence elements in the upper stem domain of vertebrate Y RNAs impair their function in DNA replication (Gardiner et al., 2009; Wang et al., 2014). As this domain is conserved in sbRNAs (Fig. 1), we synthesised mutant CeN133 and CeN135 RNAs that have base substitutions spanning the highly conserved central UG-CA tetra-nucleotide motif in the upper stem domain (mt US RNAs) (Fig. 3A,B). sbRNAs and vertebrate Y RNAs also have a conserved penta-nucleotide motif at the 5′ end of the loop domain (Fig. 1), typically UUA(U/C)C (Boria et al., 2010). We therefore synthesised mutant CeN133 and CeN135 RNAs that have base substitutions in this motif (mt LM RNAs) (Fig. 3A,B). The US and LM mutants of both CeN133 and CeN135 RNAs did not significantly increase the proportions of replicating nuclei above background levels (Student's *t-*test, *P*>0.05), indicating that their activity is severely compromised (Fig. 3C). We conclude that the upper stem domain and the conserved loop motif are important for the function of sbRNAs in DNA replication *in vitro*.

The upper stem domain of vertebrate Y RNAs is sufficient for their function (Gardiner et al., 2009; Wang et al., 2014). To test whether this is also true for nematode sbRNAs, we synthesised the corresponding domains of CeN133 and CeN135 sbRNAs (Fig. 3A,B), either with a short polypyrimidine loop (US), or with the pentanucleotide motif of the corresponding 5′ loop sequences (USLM). Both short upper stem RNAs were as active as the full-length sbRNAs (Fig. 3C), indicating that the upper stem domain of sbRNAs is also functionally sufficient. Furthermore, the conserved single-stranded loop motif is not essential in the context of the small stem RNAs (Fig. 3C) and its function therefore appears restricted to the full-length sbRNAs.

### Endogenous small RNAs from *C. elegans* are functionally active

**Fig. 4.**
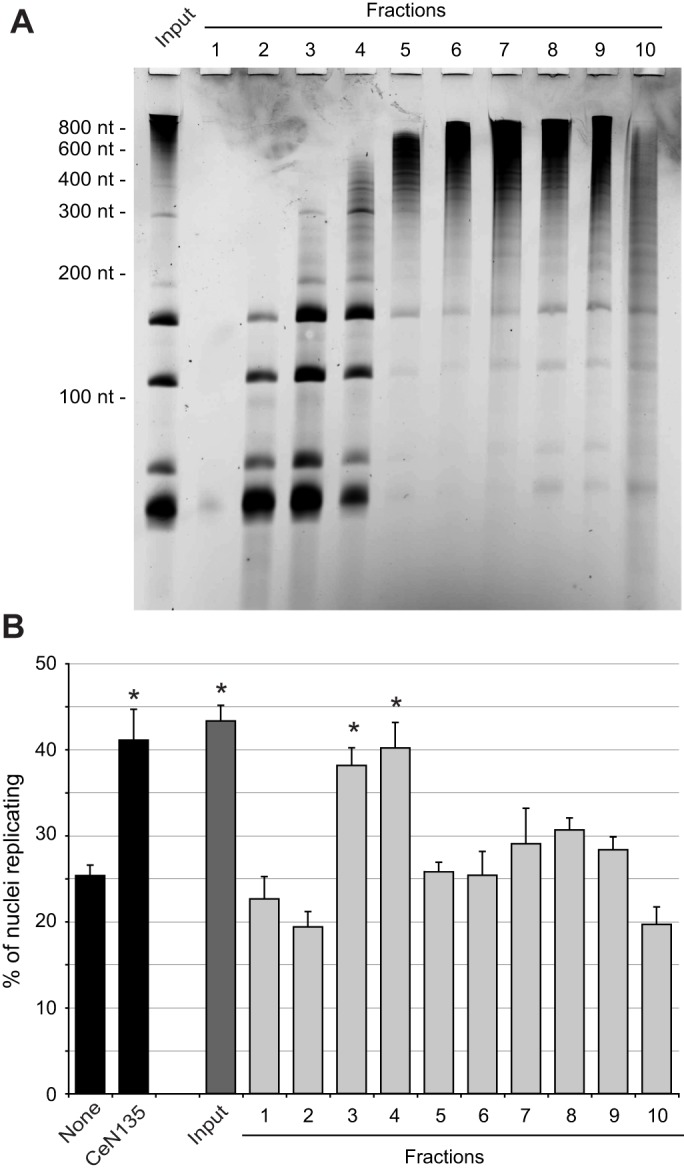
**Isolation of functionally active endogenous RNAs from *C. elegans*.** Total RNA was isolated from *C. elegans* embryos (input), and separated according to molecular mass by ultracentrifugation through a sucrose gradient (fractions 1 to 10). (A) Visualisation of endogenous RNA by denaturing PAGE and staining with SYBR-Gold. Positions of RNA molecular mass markers are indicated. (B) Functional testing *in vitro*. The indicated endogenous RNAs were tested alongside CeN135 sbRNA, as a positive control, for their ability to initiate DNA replication as detailed for Fig. 2C (mean±s.e.m.; *n*=3). *P<0.05 when compared to background level with no RNA added, as determined by Student's *t*-tests.

We next tested whether endogenous small RNAs of *C. elegans* could initiate DNA replication *in vitro*. Using the same total RNA from *C. elegans* embryos that we employed for the expression analysis (see Fig. 2A), we then fractionated this RNA by molecular mass (Fig. 4A), and tested whether or not endogenous RNAs were able to substitute for Y RNAs in the cell-free DNA replication initiation system (Fig. 4B). Indeed, fractions of small endogenous RNAs were as active as exogenous CeN135 RNA (Fig. 4B). The molecular mass of the active endogenous RNA corresponds to ∼80–300 nucleotides, which includes the size range of sbRNAs. Therefore, we next investigated the activity of sbRNAs *in vivo*.

### Functional inactivation of sbRNAs in *C. elegans* using antisense morpholino oligonucleotides

In order to probe the endogenous role of sbRNAs we needed to perturb their function in live worms. There are 19 sbRNAs in *C. elegans,* several of which can support initiation of DNA replication *in vitro,* suggesting that they might act redundantly *in vivo* (Fig. 2). Therefore, it is not straightforward to deplete sbRNA function by RNA interference or gene deletion as one might need to target many, or all of them, concurrently. We decided to target sbRNA function using antisense MOs, which we have previously used to inhibit Y RNA function in *Xenopus* and zebrafish (Collart et al., 2011). Importantly, in these cases, we found that Y-RNA–MO complexes form dominant-negative inhibitors of DNA replication, so that non-targeted Y RNAs are unable to rescue the phenotypes resulting from MO-inactivation of a targeted, but functionally redundant, Y RNA (Collart et al., 2011). We designed antisense MOs against unique regions on the six *C. elegans* sbRNAs that showed DNA replication initiation activity *in vitro* (supplementary material Fig. S2). To control for off-target effects, we used a control MO (coMO) directed against a sequence absent from the *C. elegans* genome, and used in previous studies in nematodes (Louvet-Vallée et al., 2003; Zheng et al., 2005).

**Fig. 5.**
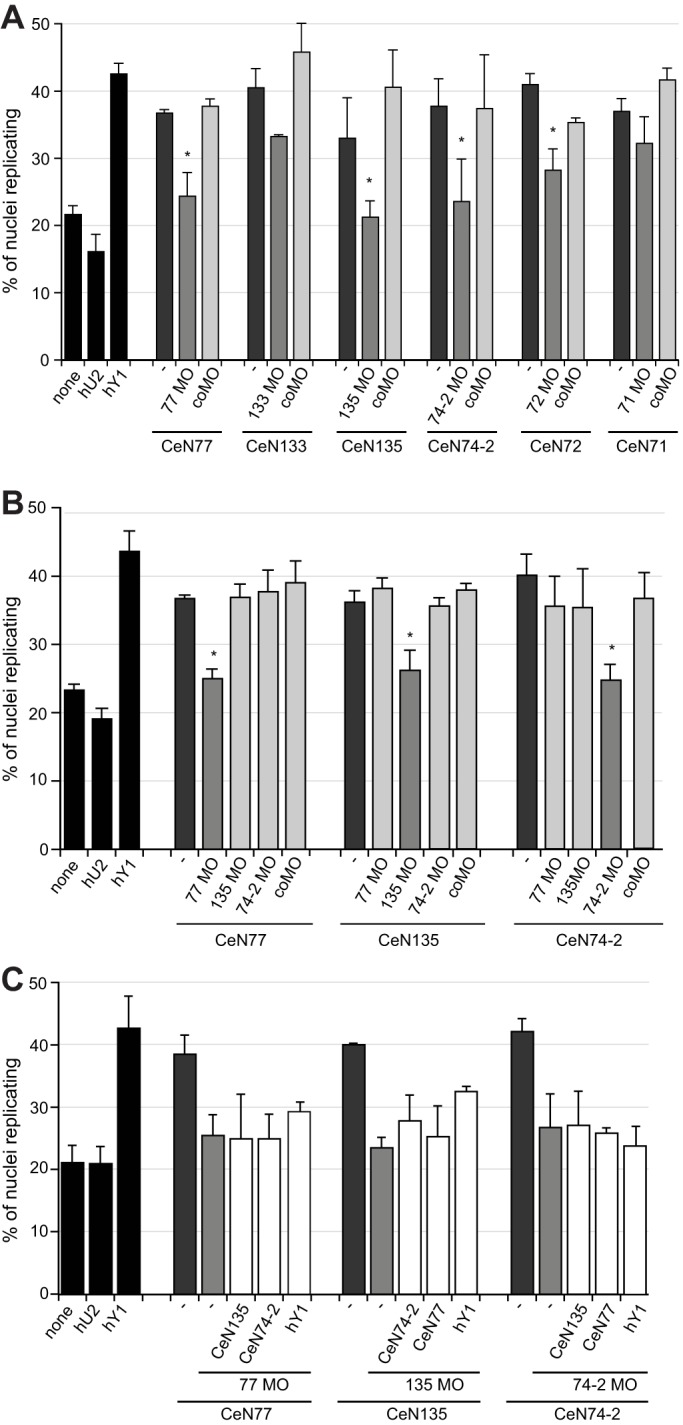
**Antisense MOs specifically inhibit sbRNA function *in vitro*.** Template nuclei from late G1 phase cells were incubated with protein fractions QA and ArFT, the indicated RNAs and MOs. (A) MOs inhibit their cognate sbRNA function. sbRNAs were either tested in the absence of any MO (–), or in the presence of their complementary MO or the control MO (coMO). (B) MOs inhibit sbRNA function specifically. sbRNAs were tested without MO (–), with their complementary MO, with non-complementary MOs or with coMO. (C) sbRNA–MO complexes are dominant-negative inhibitors of DNA replication *in vitro*. sbRNAs were tested with their complementary MO in the additional presence of the indicated non-target sbRNAs or human Y1 (hY1) RNA. Proportions of replicating nuclei were determined as detailed for Fig. 2C. Mean±s.e.m. values are shown. *n*=7 (A); *n*=5 (B); *n*=4 (C). *P<0.05 when compared to sbRNAs tested without MO (–).

First, we tested whether the MOs affect the DNA replication initiation activity of their complementary sbRNAs in the human cell-free system (Fig. 5). sbRNAs supported the initiation of DNA replication, and the addition of coMO did not inhibit this activity (Fig. 5A). In contrast, addition of complementary MOs decreased the proportion of nuclei replicating in all cases. MOs against CeN77, CeN135, CeN74-2 and CeN72 were most efficient, resulting in a significant decrease in the proportion of nuclei replicating (Student's *t-*tests, *P*<0.05, Fig. 5A). Thus, sbRNA-specific MOs target their complementary sbRNAs and inactivate their function in the initiation of DNA replication *in vitro*.

MOs can have non-specific off-target effects (Eisen and Smith, 2008). We therefore assessed the specificity of the sbRNA MOs by testing whether they inhibited non-targeted sbRNAs *in vitro*. We focused on the three sbRNAs that were most effectively inactivated by MOs. We tested each sbRNA in the presence of its complementary MO and the control MO as above, and also in the presence of the two non-complementary MOs (Fig. 5B). In all cases, only the complementary MO significantly inhibited the initiation of DNA replication mediated by the sbRNA (Student's *t-*tests, *P*<0.05, Fig. 5B). Therefore, the three MOs used here specifically inhibit the function of their target sbRNAs.

Next, we asked whether sbRNA-MO complexes could form dominant-negative inhibitors of DNA replication, as we have reported previously for vertebrate Y-RNA–MO complexes (Collart et al., 2011). We combined sbRNAs with their complementary MO and also supplemented the reactions with non-targeted sbRNAs or human Y1 RNA. The non-targeted RNAs were unable to rescue the inhibition effected by the complementary MO (Fig. 5C). We conclude that sbRNA-MO complexes are dominant-negative inhibitors of DNA replication, which cannot be overcome by an excess of functionally equivalent sbRNAs.

### sbRNAs are essential for embryonic development and viability of *C. elegans*

**Fig. 6.**
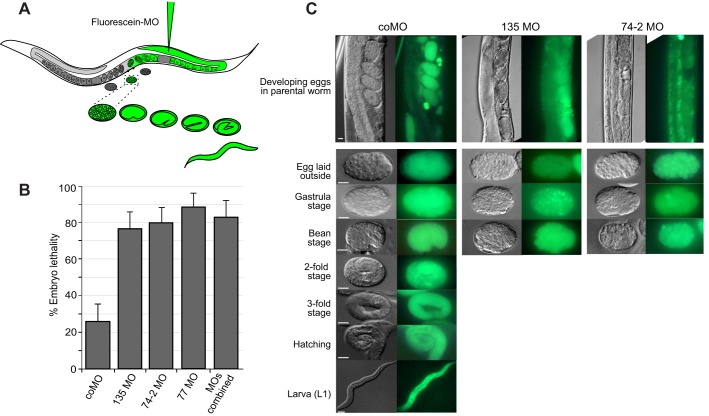
**Functional inhibition of sbRNAs by antisense MOs results in embryonic lethality in *C. elegans.*** (A) Schematic drawing of the experimental approach. Fluorescein-conjugated MOs are injected into the syncytial gonad of an adult worm. Thus, the presence of injected MOs can be traced during *in* *utero* and *ex utero* development. (B) Embryo lethality after injection of MOs. Young adult N2 worms were injected with fluorescein-conjugated coMO, or a mix of the coMO with non-fluorescent MOs targeting the individual sbRNAs CeN135, CeN74-2 or CeN77, or a combination of these three MOs. Embryo lethality was scored as the percentage of unhatched eggs relative to the total number of fluorescein-labelled eggs. Mean±s.e.m. values are shown, with *n*=5–7 and over 130 embryos analysed each time. (C) Phenotypes of developing embryos after MO injection. Young adult N2 worms were injected with fluorescein-conjugated MOs as indicated. Development was followed over time using DIC and fluorescence microscopy. Representative embryos at different developmental stages are shown. Scale bars: 10 µm.

We next used MOs to inactivate sbRNAs *in vivo*. We microinjected the syncytial gonads of adult wild-type N2 worms with MOs and investigated the phenotypic consequences (Fig. 6A). In order to trace the presence of MOs during development, we coinjected the sbRNA-targeting MOs with fluorescein-conjugated coMO. Within 4 h following injection, embryos had incorporated the MOs, as judged by green fluorescence. Following injection with coMO, ∼75% of labelled embryos hatched within 18 h whereas ∼25% died (Fig. 6B). In contrast, following injection with MOs targeting CeN77, CeN135 or CeN74-2 alone or all three combined, only ∼20% of the embryos hatched whereas ∼80% died (Fig. 6B). Examination of the dead embryos showed that following injection of MOs against CeN135 or CeN74-2, the majority of labelled embryos arrested early in development at or before the bean stage (Fig. 6C).

**Fig. 7.**
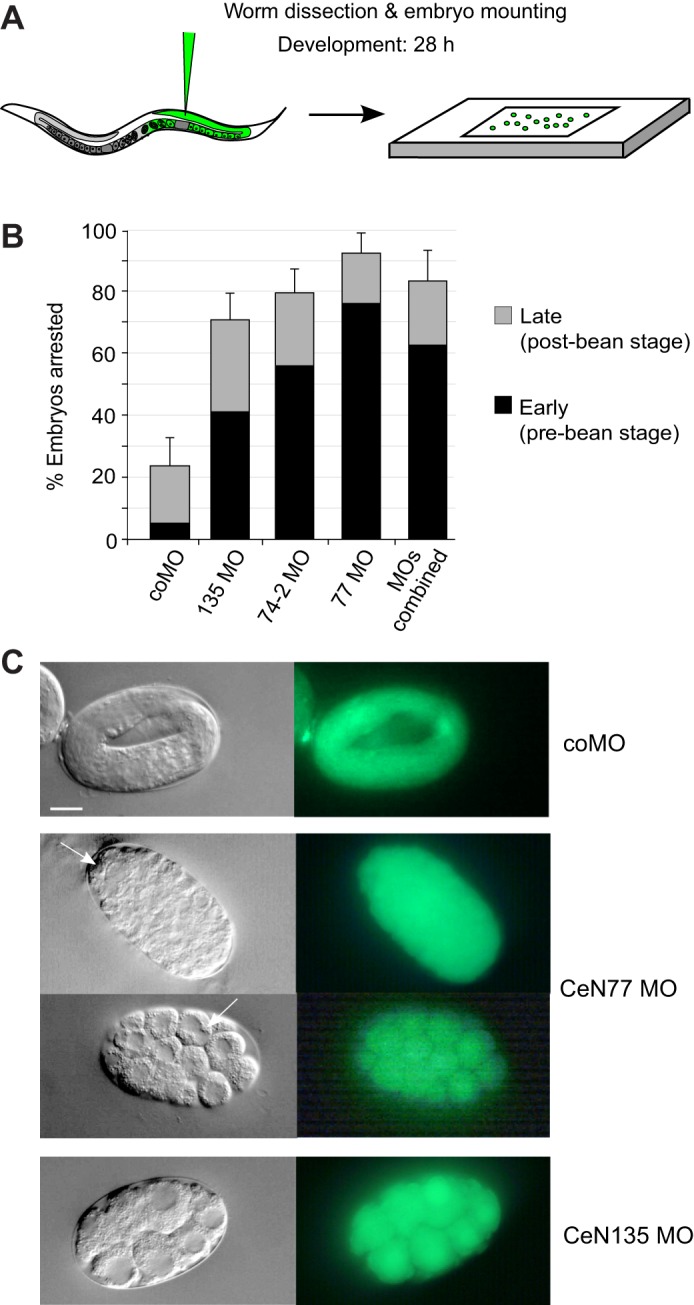
**Functional inhibition of sbRNAs results in early embryonic arrest.** Terminal phenotype analysis. (A) Schematic drawing of the experimental layout. (B) Quantification of terminal phenotypes. DIC microscopy was used to determine whether embryos injected with the indicated MOs arrested early or late (i.e. before or after the bean stage of development). Mean±s.e.m. values are shown, with *n*=6–7 and over 130 embryos analysed each time. (C) Phenotypes of arrested embryos. Representative images obtained by DIC (left) and fluorescence microscopy (right). Arrows indicate cells that have failed to separate during cell division. Scale bars: 10 µm.

To examine this lethality in a more detailed and controlled way we performed terminal phenotype analysis (Fig. 7A). Young adult worms were injected with MOs, dissected, and their embryos mounted onto agar pads. Embryos were allowed to develop until they reached their terminal phenotype and were then imaged and scored. This assay thereby avoids any variability in egg laying and plate conditions. We scored embryos as early-arresting if their terminal phenotype occurred at or before the bean stage (the stage at which morphogenesis becomes apparent), which covers the period of bulk cell proliferation and gastrulation during *C. elegans* development (Sulston et al., 1983). We scored embryos as late-arresting if they failed to hatch and their terminal phenotype occurred after the bean stage. Following microinjection of coMO, ∼20% of embryos failed to hatch and of these, ∼5% showed early-arresting terminal phenotypes (Fig. 7B). In contrast, microinjection of MOs targeting CeN77, CeN135 or CeN74-2 resulted in 70–92% arrested embryos, of which 40–73% showed early arrest (Fig. 7B). Microinjection of all three MOs combined also resulted in over 80% lethality and ∼60% early-arrested embryos (Fig. 7B). Early-arresting embryos contained abnormally large undifferentiated cells, multinucleated cells and cells that were stuck in the cleavage phase of cytokinesis (Fig. 7C). These phenotypes likely reflect defects in chromosome segregation and are characteristic of DNA replication mutants in *C. elegans* (Brauchle et al., 2003; Encalada et al., 2000; Gaggioli et al., 2014). The proportion of late-arresting embryos (∼20%) is not affected by the sequence of the MOs used and so is most likely due to a non-specific effect. Taken together, these data indicate that sbRNAs are essential for viability and early embryonic development of *C. elegans*.

### Functional inactivation of sbRNAs *in vivo* results in S phase defects in early *C. elegans* embryos

**Fig. 8.**
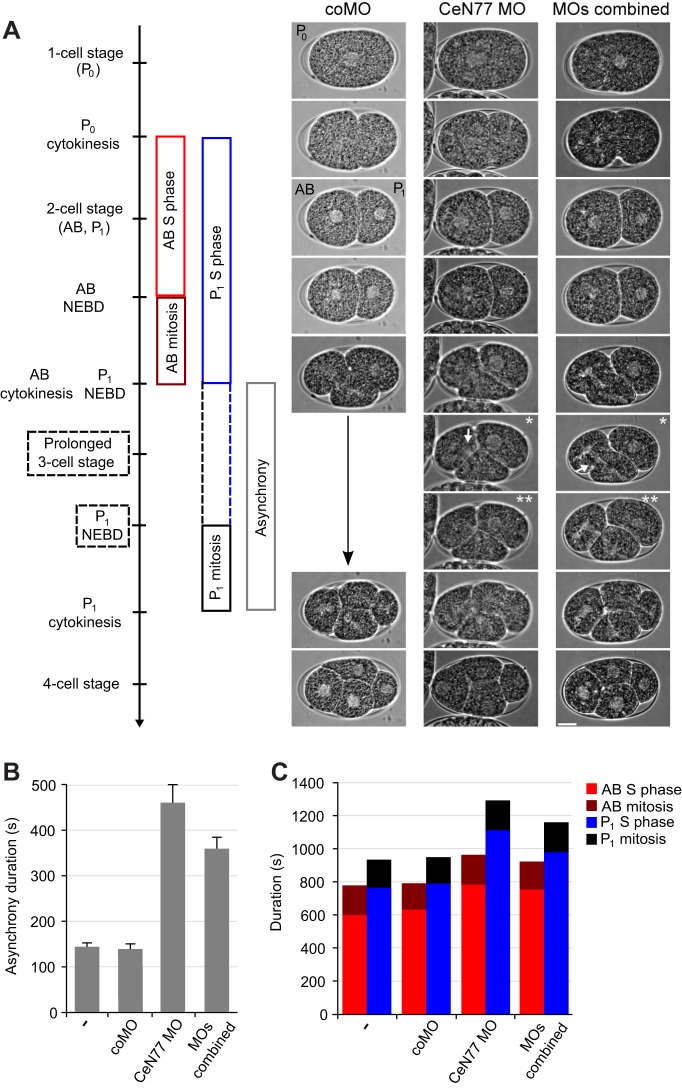
**Functional inhibition of sbRNAs lengthens S phase and the asynchrony of AB and P_1_ blastomere division in early embryos.** (A) Representative time-lapse DIC images of one-cell–four-cell stage embryos isolated from N2 worms microinjected with the indicated MOs. MOs combined, MOs targeting CeN135, CeN74-2 and CeN77 sbRNAs. Key stages of cell divisions during embryonic development are indicated on the left. Prolonged and delayed stages are indicated by dashed lines. All embryos are shown with the anterior side and the AB cell on the left. NEBD, nuclear envelope breakdown. A single white asterisk indicates embryos with prolonged and persistent three-cell stages due to the increased time interval between AB and P_1_ divisions. Two white asterisks indicate embryos where a prolonged three-cell stage results in a delayed P_1_ NEBD after the AB blastomere has divided. The white arrow indicates chromosome bridges. Scale bar: 10 µm. (B,C) Quantification of the durations of asynchrony (B), and S phase and mitosis in AB and P_1_ blastomeres (C). Embryos were analysed in the absence of MO (–) or the presence of the specified MOs. Full numerical values are given in supplementary material Table S1.

To further dissect the role of sbRNAs in cell cycle progression, we analysed embryos undergoing the first three mitotic cell cycles, when defects are more clearly detectable (Benkemoun et al., 2014; Brauchle et al., 2003; Encalada et al., 2000; Gaggioli et al., 2014). In early *C. elegans* embryos, the cells divide asynchronously and the cell cycles consist only of alternating S phase and mitosis, with no intervening gap phases (Edgar and McGhee, 1988). In the first mitotic division, the P_0_ blastomere divides asymmetrically to generate an anterior blastomere, AB, and a smaller posterior blastomere, P_1_ (Fig. 8A). These cells have different developmental fates and cell division timing, so that AB has a shorter S phase and divides shortly before P_1_ (Brauchle et al., 2003; Edgar and McGhee, 1988) (Fig. 8A).

In wild-type or coMO-loaded embryos, P_1_ divided ∼150 s after AB (Fig. 8B; supplementary material Table S1), which is consistent with previous reports (Benkemoun et al., 2014; Brauchle et al., 2003; Encalada et al., 2000; Gaggioli et al., 2014). However, in embryos loaded with MOs against CeN77 or with a cocktail of three MOs against CeN77, CeN135 and CeN74-2 the duration of this cell cycle asynchrony increased up to threefold, to ∼450 s and ∼360 s, respectively (Fig. 8B; supplementary material Table S1). This delay in P_1_ division resulted in a prominent and prolonged three-cell stage (Fig. 8A,B; supplementary material Table S1). Notably, these embryos also contained chromosome bridges (Fig. 8A), indicative of chromosome segregation defects. These phenotypes are all characteristic of DNA replication mutants, in which defects in chromosome segregation arise, presumably due to incomplete DNA replication (Benkemoun et al., 2014; Brauchle et al., 2003; Encalada et al., 2000; Gaggioli et al., 2014).

To determine more precisely which stages of the cell cycles were affected in the embryos, we measured the duration of S phase and mitosis in the AB and P_1_ blastomeres (Fig. 8A,C). The duration of mitosis was not significantly affected by any MOs under these conditions (Fig. 8C; supplementary material Table S1). In contrast, the progression through S phase was significantly delayed in both AB and P_1_ cells in the embryos containing the sbRNA-specific MOs, compared to wild-type and coMO-containing embryos (Fig. 8C; supplementary material Table S1). As reported previously for defects in DNA replication machinery, the S phase of P_1_ was particularly delayed (Benkemoun et al., 2014; Brauchle et al., 2003; Encalada et al., 2000; Gaggioli et al., 2014). Therefore, the long P_1_ cell division delay in these embryos is due directly to a delay in S phase progression, but not mitosis.

Taken together, our *in vivo* data (Figs 6–8) indicate that sbRNAs are required for early embryonic development in *C. elegans*. Functionally inactivating sbRNAs results in phenotypes similar to those of DNA replication mutants, including delayed S phase progression and chromosomal bridges, as well as developmental arrest during the period that correlates with bulk cell proliferation in *C. elegans* embryos.

## DISCUSSION

sbRNAs were recently identified as a family of small non-coding RNAs in nematodes (Aftab et al., 2008; Boria et al., 2010; Deng et al., 2006). The existence of conserved nucleotide sequence elements and structural motifs suggests that sbRNAs might be homologues of vertebrate Y RNAs (Boria et al., 2010). In this study, we report the first functional characterisation of sbRNAs. We have shown that several sbRNAs are functionally equivalent to vertebrate Y RNAs and can support the initiation of chromosomal DNA replication *in vitro*. Inactivation of sbRNA function *in vivo* results in embryonic lethality and DNA replication defects during early embryogenesis in *C. elegans*. We have therefore uncovered a previously unknown functional link between non-coding sbRNAs and the regulation of cell proliferation in *C. elegans.* Taken together, our findings strongly support the view that sbRNAs are functional homologues of vertebrate Y RNAs.

### sbRNAs support initiation of DNA replication *in vitro*

Several nematode sbRNAs are able to support efficient initiation of DNA replication in a human cell-free system and act redundantly with each other. As for Y RNAs (Gardiner et al., 2009; Wang et al., 2014), a conserved structural motif in the upper stem of sbRNA is essential and sufficient for this activity. Whereas all Y RNAs tested thus far were active (Christov et al., 2006; Gardiner et al., 2009), the situation appears more complex in the larger group of nematode sbRNAs. Two *C. elegans* sbRNAs, namely CeN73-1 and CeN76, did not show significant DNA replication initiation activity despite the presence of the conserved nucleotide sequences in their upper stem domain (Fig. 2C). This suggests that although the upper stem is essential and sufficient to provide sbRNA function as a small isolated RNA domain (Fig. 3), it might not be sufficient in the context of some full-length RNAs in this system. In support of this scenario, the adjacent loop domain might also play an essential supportive role in sbRNA function *in vitro*, because mutations of the highly conserved short sequence motif at the 5′ end of the loop abrogated the activity of full-length sbRNA *in vitro* (Fig. 3). A further possibility is that the heterogeneously sized and highly divergent single-stranded loop domains of some sbRNAs might mediate non-specific molecular interactions with human proteins present in the cell-free system, which could lead to non-specific steric inhibition. Alternatively, there could be additional unknown cis-acting determinants, shared only by the active sbRNAs, which contribute to sbRNA functionality *in vitro*. Systematic mutagenesis would be required to dissect the roles of the structural domains of sbRNAs further.

### sbRNA function is essential *in vivo*

We probed the function of sbRNAs *in vivo* by functionally inactivating them in developing *C. elegans* embryos. There are 19 sbRNAs in *C. elegans*, some of which are functionally equivalent *in vitro* (Fig. 2C). It is therefore difficult to achieve sbRNA depletion by RNA interference or gene deletion, as several or all of the sbRNAs might need to be targeted together. Indeed, *C. elegans* carrying a single deletion in CeY show no deleterious phenotypes (Boria et al., 2010), and we observed no obvious phenotypes in a *C. elegans* strain (VC30032) carrying a point mutation in the upper stem of CeN74-2 (*gk406084*). We therefore targeted sbRNA function using a dominant-negative approach utilising MOs, which we have previously used successfully to inhibit Y RNA function in *Xenopus* and zebrafish (Collart et al., 2011). The dominant-negative phenotype most likely arises because complexes of MOs and Y RNAs lock down interactions of Y RNAs with initiation proteins or chromatin in an inactive state (Collart et al., 2011). MOs have been used to inhibit gene expression in other nematode species (Louvet-Vallée et al., 2003; Zheng et al., 2005), and we have established here that MOs can specifically inactivate the function of non-coding sbRNAs directly. Our data should therefore enable and encourage the future use of MOs as a powerful antisense oligonucleotide tool to inactivate functionally redundant non-coding RNAs in *C. elegans*. An additional advantage that this approach offers is that it can inactivate additional redundant non-coding RNAs acting in the same pathway that might not have been discovered at the time of analysis, and which would thus escape genetic approaches that target known candidate genes. Nevertheless, it remains a formal caveat of this current study that we have not tested deletions of sbRNAs.

Functional inactivation of sbRNAs by MO injection into the syncytial gonad of *C. elegans* resulted in high levels of embryonic arrest during the early period of bulk cell proliferation (Sulston et al., 1983). These early-arresting embryos also showed characteristic DNA replication defects, including large undifferentiated cells and chromosome segregation defects, such as chromosomal bridges and multinucleated cells (Fig. 7C; Fig. 8A). Inactivation of sbRNAs also resulted in a delay in S phase progression in 2–4-cell stage embryos, but the duration of mitosis was not affected. Collectively, these phenotypes are all hallmarks of DNA replication mutants (Benkemoun et al., 2014; Brauchle et al., 2003; Encalada et al., 2000; Gaggioli et al., 2014), and taken together with our *in vitro* data, strongly support a functional role for sbRNAs during DNA replication.

Inactivation of Y RNAs and sbRNAs in developing *Xenopus*, zebrafish and *C. elegans* embryos, respectively, results in broadly similar phenotypes, but the timing and onset of these phenotypes is different. This could be due to differences in the developmental regulation of cell proliferation in the early embryos of these organisms. In *Xenopus* and zebrafish, Y RNAs are not required for DNA replication before the mid-blastula transition (MBT) (Collart et al., 2011), when cell cycles are synchronous and alternate between S phase and mitosis, without intervening gap phases (Newport and Kirschner, 1982a). After the MBT, Y RNAs become essential for DNA replication and viability (Collart et al., 2011). This corresponds to the onset of asynchronous cell cycles with gap phases, site-specific initiation of DNA replication and bulk zygotic transcription (Brown and Littna, 1964; Hyrien et al., 1995; Langley et al., 2014; Newport and Kirschner, 1982b). The current consensus is that *C. elegans* does not undergo such a clearly defined MBT (van den Heuvel, 2005). Early cell cycles in the *C. elegans* embryo also lack gap phases but are asynchronous and asymmetric, with the first five divisions giving rise to the founder cells (AB, MS, E, C, D and P4) (Edgar and McGhee, 1988; Sulston et al., 1983). The descendants of these founder cells, which produce defined cell lineages and tissue types, undergo mostly synchronous cell divisions and include gap phases, but the transition to this cell cycle profile varies in each cell lineage (Sulston et al., 1983; van den Heuvel, 2005). Zygotic transcription in *C. elegans* undergoes a more defined transition: major zygotic transcription begins at approximately the 30-cell stage, although minor transcription already occurs in the 2–4-cell stage embryo (Baugh et al., 2003). Upon sbRNA inactivation, S phase defects are already apparent during the first two to three embryonic cell divisions (Fig. 8), although most embryos undergo several more cell cycles before arresting (Fig. 7). This is consistent with previous studies that have disrupted components of the DNA replication machinery in *C. elegans* (Benkemoun et al., 2014; Brauchle et al., 2003; Encalada et al., 2000; Gaggioli et al., 2014). Taken together, sbRNAs, like Y RNAs in *Xenopus* and zebrafish, appear to be important players in the developmental regulation of cell proliferation in the early embryo, but the execution point for the function of these non-coding RNAs is different, possibly reflecting the differences in cell cycle timing, cell fate definition or differentiation in the development of these animals.

### Interaction of sbRNAs with replication proteins

There is currently no homologous cell-free system available to study the regulation of nematode DNA replication using cell extracts from *C. elegans*. We therefore used an established human cell-free system. Our data indicate that sbRNAs can interact with human DNA replication proteins and form functionally active complexes that support the initiation of DNA replication *in vitro*. The molecular mechanisms underpinning sbRNA function in *C. elegans* have not yet been identified, but it is likely that sbRNAs exert their function by interacting with components of the nematode DNA replication initiation machinery *in vivo*. In vertebrates, the homologous Y RNAs interact with proteins essential for the initiation of DNA replication, including the origin recognition complex (ORC), Cdc6 and Cdt1 (Collart et al., 2011; Zhang et al., 2011). In *Xenopus*, the ORC is required for Y RNAs to associate with chromatin after the MBT (Collart et al., 2011), and other non-coding RNAs have been shown to interact with the ORC in other organisms (Mohammad et al., 2007; Norseen et al., 2008). In *C. elegans*, the ORC1–ORC5 subunits are involved in DNA replication origin licensing (Sonneville et al., 2012). The redundant activities of sbRNAs and Y RNAs in DNA replication *in vitro*, and their dominant-negative inhibition by antisense MOs therefore suggest that there is a common pathway for the action of these RNAs. It is possible that any one sbRNA or Y RNA is recruited to sites of DNA replication initiation in an ORC-dependent manner to execute their as yet unknown biochemical function. A recent solution-state structural analysis of the conserved upper stem domain of human Y1 RNA suggests that it might activate unknown target proteins through an allosteric mechanism (Wang et al., 2014). Addition of dominant-negative MOs would lock this RNA-containing complex in an inactive state and thus render the initiation site incapable of initiation of DNA replication and also block interaction with other active RNAs. It will therefore be of great interest to investigate if and how sbRNAs associate with the ORC or other DNA replication proteins in nematodes, and how far these interactions are conserved between nematodes and vertebrates.

To conclude, our results indicate that sbRNAs are required for embryonic development, cell proliferation and S phase progression in *C. elegans*. These findings reveal that the regulation of metazoan DNA replication by small non-coding RNAs spans across the animal kingdom, and could be a conserved and fundamental principle.

## MATERIALS AND METHODS

### Bioinformatics

sbRNA sequences, as previously published (Boria et al., 2010), are listed in supplementary material Table S2. RNA secondary structures were predicted using Mfold v.3.6 (http://mfold.rna.albany.edu) under default conditions (Mathews et al., 1999; Zuker, 2003). Results are displayed using LocARNA (Will et al., 2007) using an online tool (http://rna.informatik.uni-freiburg.de). Full-length sbRNA sequences were aligned manually. WebLogos (Crooks et al., 2004) were generated using an online tool (http://weblogo.berkeley.edu) from nine base-pairs in the upper stem and the penta-nucleotide motif in the loop.

### *C. elegans* culture, RNA preparation and determination of sbRNA expression levels

The *C. elegans* N2 strain was maintained according to standard methods (Lewis and Fleming, 1995). Embryos were prepared by treatment with alkaline hyopchlorite. For L4 larvae embryos were allowed to hatch in the absence of food, the resulting synchronised L1 larvae were then plated onto food and grown to L4 (Lewis and Fleming, 1995).

Total RNA was isolated by using TRIzol reagent according to the manufacturer's instructions (Life Technologies) with the addition of ten freeze-thaw cycles and LiCl precipitation (Collart et al., 2011). Total RNA was fractionated according to size by sedimentation on linear 15–40% sucrose gradients prepared in replication buffer (20 mM K-HEPES pH 7.8, 100 mM K acetate, 1 mM DTT, 0.5 mM EGTA). Gradients were centrifuged in a Beckman Coulter MLS50 rotor at 124,000 ***g*** for 18 h at 4°C.

cDNA was synthesised from total RNA preparations using random hexamer primers (Promega) with the SuperScript II Reverse Transcriptase kit according to the manufacturer's protocol (Invitrogen). cDNA was used as a template for quantitative RT-PCR on the iCycler iQ platform, using the KAPA SYBR FAST qPCR master mix (KAPA Biosystems) over 45 cycles and a hybridisation temperature of 55°C (Christov et al., 2006). Specific primer pairs for each sbRNA are listed in supplementary material Table S3. The amount of each sbRNA relative to the overall mean amount of all sbRNAs combined was calculated from the threshold cycles (CT) of each cDNA amplification. For each primer pair, the individual CT values were first normalised by subtracting the CT value of a control reaction using genomic DNA from the CT values of the cDNA amplification. Relative ΔCT values were then calculated using the following equation: ΔCT=CT_sbRNA_ – CT_mean_, where CT_mean_ is the mean of all individual CT_sbRNA_ values.

### Synthesis and purification of wild-type and mutant RNAs

Recombinant cDNA template sequences for expression of sbRNAs and their mutant derivatives were generated by PCR and TOPO TA cloning (Invitrogen), as described previously (Christov et al., 2006; Gardiner et al., 2009). Templates for human Y1 and U2 RNAs were generated previously (Christov et al., 2006). Sequences of the oligonucleotides used are listed in supplementary material Tables S4 and S5.

Individual RNAs were synthesised from these templates by *in vitro* transcription using SP6 RNA polymerase (Christov et al., 2006; Gardiner et al., 2009). RNAs were purified by anion exchange chromatography (Zhang et al., 2011). The size and purity of all *in vitro* synthesised RNA was confirmed by 8 M urea denaturing polyacrylamide gel electrophoresis and staining with SYBR Gold (Invitrogen). Multimeric 100-nucleotide RNA molecules (Fermentas) were used as molecular mass markers.

### *In vitro* DNA replication assays

*In vitro* DNA replication assays were performed as described previously (Christov et al., 2006; Gardiner et al., 2009). Template nuclei were isolated from human EJ30 bladder carcinoma cells synchronised in late G1 phase by 0.6 mM mimosine (Krude, 2000). Cytosolic extract from proliferating human HeLa cells was obtained from Cilbiotech (Mons, Belgium) and fractionated by anion exchange chromatography into protein fractions QA and ArFT (Christov et al., 2006). Reactions contained template nuclei and digoxigenin-11-dUTP (Roche), as a tracer, together with 7 µg of each protein fractions QA and ArFT in a reaction volume of 50 µl (Collart et al., 2011). For functional testing, purified RNAs were added at 170 nM and MOs at 700 nM. Nuclei were fixed after 2.5 h at 37°C and spun onto polylysine-coated glass coverslips. Digoxigenin-labelled replicated DNA was detected by anti-digoxigenin fluorescein-conjugated F_ab_ fragments (Roche), and total DNA was counter-stained with propidium iodide (Christov et al., 2006; Krude, 2000). Nuclei were visualised using confocal fluorescence microscopy performed on a SP1 Leica microscope with a 40× objective lens. Percentages of replicating nuclei were determined per reaction.

### Morpholino microinjection in *C. elegans*

Sequences of all MOs (obtained from GeneTools, LLC) are listed in supplementary material Table S6. MOs were diluted, heated for 7 min at 65°C and insoluble residues were pelleted. MOs were injected at 0.35 mM, coMO-3′-carboxyfluorescein was used as a tracer and was co-injected at 0.05 mM with other MOs. MOs were microinjected bilaterally into the syncytial gonads of young adult worms (Mello and Fire, 1995) using a Zeiss Axiovert S100 inverted microscope and an Eppendorf transjector.

### Analysis of embryonic lethality

Following microinjection with MOs, adult worms were allowed to recover for 8–10 h at 20°C, during which time non-fluorescent eggs (those already *in situ* prior to microinjection) were laid. Healthy adults were transferred onto fresh plates with food and left for 10 h at 20°C. The injected adults were then removed and the plates scored for fluorescent F1 eggs. After a further 18 h, fluorescent unhatched eggs and larvae were counted to quantify embryo lethality. The development of MO-containing eggs was monitored by removing laid eggs at regular intervals and mounting them on 3% agar pads (Walston and Hardin, 2010).

For terminal phenotype assays, injected worms were allowed to recover for 8–10 h, then dissected to release the embryos (Walston and Hardin, 2010). Embryos were mounted, allowed to develop at 15°C for at least 28 h and then imaged as above.

Embryos were imaged at room temperature with fluorescence and Nomarski differential interference contrast (DIC) microscopy using a Zeiss Axioskop2 plus microscope with a 40× objective lens, fitted with a QImaging MicroPublisher 5.0 RTV camera. Images were acquired using QCapture Pro software and processed using GIMP Image Editor.

### Time-lapse imaging of embryonic development

For AB–P_1_ division measurements, embryos were obtained by dissecting worms microinjected with MOs and then mounted in egg buffer (Edgar, 1995). Live imaging was performed at room temperature at 18–28 h after microinjection, with a Leica SP5 inverted scanning laser microscope and 63×1.2 NA Olympus water immersion objective, using DIC settings. Images were acquired with Leica LAS AF imaging software as *z*-stacks of nine levels taken every ∼12 s for durations that covered the time from P_0_ cytokinesis until the four-cell stage. Images were processed using ImageJ. Timing of nuclear envelope breakdown (NEBD) (measured at disappearance of nuclear membranes) and cytokinesis (measured at onset of cortical furrowing) in P_0_, AB and P_1_ blastomeres was determined as described previously (Benkemoun et al., 2014; Brauchle et al., 2003; Edgar and McGhee, 1988).
